# Predictors of retention in the prospective HIV prevention OKAPI cohort in Kinshasa

**DOI:** 10.1038/s41598-021-84839-w

**Published:** 2021-03-08

**Authors:** S. Carlos, E. Burgueño, A. Ndarabu, G. Reina, C. Lopez-del Burgo, A. Osorio, B. Makonda, J. de Irala

**Affiliations:** 1 Department of Preventive Medicine and Public Health, University of Navarra, Pamplona, Spain; 2 IdiSNA, Instituto de Investigación Sanitaria de Navarra, Pamplona, Spain; 3 Institute for Culture and Society, University of Navarra, Pamplona, Spain; 4 Faculté de Médecine, Université de Mwene-Ditu, Mwene-Ditu, Democratic Republic of the Congo; 5 Monkole Hospital/CEFA, Kinshasa, Democratic Republic of the Congo; 6 Microbiology Department, Clínica Universidad de Navarra, Pamplona, Spain; 7 School of Education and Psychology, University of Navarra, Pamplona, Spain

**Keywords:** Diseases, Risk factors

## Abstract

Retention is a key element in HIV prevention programs. In Sub-Saharan Africa most data on retention come from HIV clinical trials or people living with HIV attending HIV treatment and control programs. Data from observational cohorts are less frequent. Retention at 6-/12-month follow-up and its predictors were analyzed in OKAPI prospective cohort. From April 2016 to April 2018, 797 participants aged 15–59 years attending HIV Voluntary Counseling and Testing in Kinshasa were interviewed about HIV-related knowledge and behaviors at baseline and at 6- and 12-month follow-ups. Retention rates were 57% and 27% at 6- and 12-month follow up; 22% of participants attended both visits. Retention at 6-month was significantly associated with 12-month retention. Retention was associated with low economic status, being studying, daily/weekly Internet access, previous HIV tests and aiming to share HIV test with partner. Contrarily, perceiving a good health, living far from an antiretroviral center, daily/weekly alcohol consumption and perceiving frequent HIV information were inversely associated with retention. In conclusion, a high attrition was found among people attending HIV testing participating in a prospective cohort in Kinshasa. Considering the low retention rates and the predictors found in this study, more HIV cohort studies in Kinshasa need to be evaluated to identify local factors and strategies that could improve retention if needed.

## Introduction

Despite a 28% decline in new HIV infections in Eastern and Southern Africa from 2010 to 2018 and of 13% in Western and Central Africa, there are still 25.6 million people living with HIV in Sub-Saharan Africa (SSA), a region where 61% of new infections worldwide take place^1^. These figures show that there has been an insufficient progress in the prevention, treatment and control of HIV.

Retention at the different stages of the HIV continuum of care is a key element for the success of HIV control programs^2–10^. It can lead to a better medication adherence, enabling dosage or drug modifications, status disclosure to partner, and better social support, thus reducing the risk of antiretroviral therapy (ART) resistance and HIV transmission, morbidity and mortality^11–13^.

Retention is also essential for HIV research studies. High quality longitudinal cohorts and clinical trials within healthcare settings are necessary for the design of adequate HIV programs. Both study designs facilitate data collection, tracing the dynamics of behaviors and identify factors that influence sexual behaviors, testing or treatment adherence.

In SSA, most published research on retention and related factors show data from clinical trials or from people living with HIV within the HIV continuum of care. The problem of attrition among people living with HIV still not under ARV treatment (who have a high risk of HIV transmission) or among participants in prospective observational cohorts has been less frequently evaluated^5,14–16^.

Observational studies can show the real-world context outside the controlled protocols of clinical trials. They can help to properly design preventive interventions, and to improve their feasibility, uptake, acceptability and efficiency^17–20^. Longitudinal observational studies can also provide information concerning loss to follow-up (LTFU) and their predictors, which is relevant for the implementation of HIV preventive strategies, and to improve the quality of the data obtained^21^.

Being LTFU in longitudinal cohorts can be an important source of selection bias^22^. LTFU participants might have different characteristics from those who complete all study visits^19,23–26^. Furthermore, failing to achieve the expected number of participants can reduce the statistical power of a study^27^ and also prolong the study, modifying available economic resources^28^. Thus, retention is crucial for the validity of these studies.

Data from observational cohorts evaluating retention in ART programs in SSA have shown attritions at 12 months from 7 to 45%^10,23,29^. However, attrition rates vary for different regions and studies, being generally high in SSA^10,30,31^.

Different factors have been shown to be associated with being LTFU in the African context, including some related to the participants, their contact information, the study time, location or staff^12,32,33^. Frequent changes in cell phone numbers, transportation to study sites or financial constraints contribute largely to these figures^28^.

Research on retention in longitudinal observational studies in the Democratic Republic of the Congo (RDC) are scarce. Therefore, in this study the retention rates at 6- and 12-month follow-ups and the associated factors were analyzed in a prospective cohort study among people attending HIV testing at a reference hospital in Kinshasa (DRC).

## Methods

### Study design and participants

The OKAPI (Observational Kinshasa AIDS Prevention Initiative) project is a prospective cohort study designed to evaluate factors associated with changes in HIV knowledge and sexual behaviors after 6- and 12-months of follow-up. From April 2016 to April 2018, people aged 15–59 years attending HIV Voluntary Counseling and Testing (VCT) at a reference hospital in Kinshasa were invited to participate. Those with a previous HIV positive test as well as pregnant women were excluded. Details of the study have been published elsewhere^34^.

### Data collection

Participants were interviewed by local researchers at baseline and after 6- and 12-month follow-up periods. Male and female interviewers were available at all study times. Questionnaires were available in French and Lingala. The baseline questionnaire included 59 questions about their sociodemographics, health-related aspects, HIV knowledge, attitudes, behaviors and exposure to HIV information. Follow-up questionnaires were shorter and included some new questions concerning sociodemographic and health issues.

The sociodemographic variables considered in the analyses carried out in this paper were: sex, age (quantitative and categorized into 15–19, 20–24 and 25–59 years, for adolescents, young and adults, respectively, following the World Health Organization and other official classifications), education level (no studies/primary studies, secondary studies, university studies), perceived economic level (low, middle, high), working status (unemployed/housewife, studying, working), civil status (single, married, divorced, widowed), church attendance (never/seldom/monthly, daily/weekly), use of the mobile phone and the Internet (daily/weekly). Health-related information included: perceived health status (1 to 5 scale, categorized as 1–3 for lower health status, and 4–5 for better perceived health), HIV risk perceived (no/low risk, medium/high risk), previous HIV testing, attending HIV VCT with couple, intention to share an HIV + result with partner, time to the closest ARV center (< 15, 15–30, 30–60, > 60 min, categorized into > 60 min or not), HIV test result (negative, positive, indeterminate), STI diagnosis in the last 12 months (yes/no), diagnosis of genital ulcer, urinary tract, vaginal or penis infection (yes/no) and alcohol consumption (never/seldom, weekly/daily). With regards to sexual behaviors, participants were asked about ever sex, first sex before 15 years, ≥ 2 sexual partners currently and in the previous 6 months, consistent condom use, oral, anal or paid sex (ever) and forced sex (ever), including physical violence perpetrated by partner, sex being afraid of partner´s reaction and unwanted sexual practices. With regards to HIV misconceptions, believing HIV is caused by witchcraft or God´s punishment was considered. Finally, participants were asked about HIV information in Kinshasa (few/quite enough and much).

### HIV diagnosis

A free HIV screening test was carried out at each study visit. A blood sample was collected and rapid diagnostic tests were used, following the routine clinical protocol: Determine HIV-1/2 (Alere) test was performed and, if positive, DoubleCheckGold (Orgenics) and Unigold (Trinity Biotech) rapid immunoassays were used for confirmation. A Dried Blood Spot (DBS) card was collected for HIV molecular analyses among participants getting an HIV positive test.

### Follow-up

At baseline, each participant´s telephone number was registered, as well as up to three other contact phone numbers in order to avoid attrition. All participants received a transport fee at each follow-up appointment, as explained at recruitment. One week before each follow-up appointment, the local personnel phoned the participants up to 5 times. Participants were considered lost-to follow up (LTFU) if they did not answer to any phone-calls or they could not or did not want to remain in the study. Reasons for not coming back were recorded. Home visits were offered to LTFU participants that could not come back, in order to facilitate the HIV testing and data collection.

### Statistical analyses

Descriptive analyses were first carried out to evaluate participants´ sociodemographics, health, sexual behaviors and HIV knowledge/information at baseline and at 6- and 12-month visits. Paired comparisons between characteristics at baseline and at 6- and 12-month follow-ups, respectively, were carried out using McNemar test. Additionally, at both follow-ups characteristics of the retained and lost-to-follow-up participants were compared by using Chi2 test and Student t test for categorical and quantitative variables, respectively. Crude logistic regression analyses were carried out to evaluate the association between each baseline characteristic and retention in the cohort at 6- and 12-month appointments. A multivariate logistic regression model was fitted including all variables significantly associated with retention in the crude analyses. A different multivariate model was fitted for each follow-up time, considering the 6-, 12- or both 6- and 12-month retention as the dependent variable. An additional multivariate-adjusted model was built including time-varying exposures and a Generalized Estimated Equations (GEE) analysis was carried out for this purpose.

All p-values < 0.05 were considered statistically significant. Analyses were performed using Stata version 15.1 (StataCorp, College Station, TX, USA).

### Ethical issues

OKAPI project was approved by the Research Ethics Committees of the two centers involved in the study (University of Navarra, Spain and Monkole Hospital, DRC). A written informed consent was collected from each participant or parent and/or legal guardian if subjects were under 18. All methods were performed in accordance to the principles of the Declaration of Helsinki.

## Results

**Figure 1:**
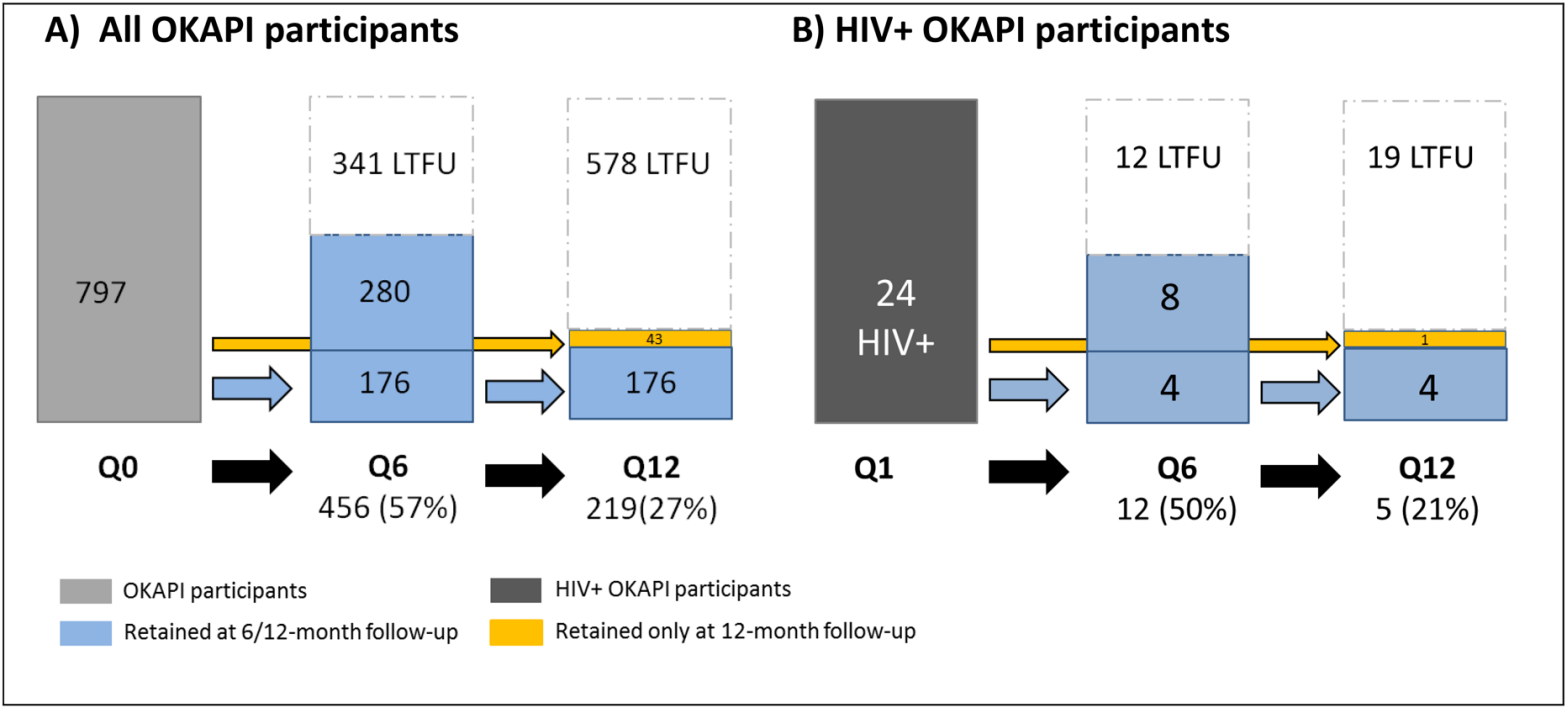
Follow-up diagram. Q0: baseline questionnaire; Q6: 6-month follow up questionnaire; Q12: 12-month follow up questionnaire; LTFU: lost-to-follow up.

From April 2016 to April 2018, 797 participants were included in the cohort. All people invited to participate agreed to join the study. All of them replied to the baseline interview and were HIV tested. At the 6-month appointment, 456/797 participants were followed up (57% retention rate): 445 (56%) returned to Monkole Hospital and 11 (1%) were interviewed and tested at home. At 12-month follow-up, 219/797 participants returned (27%). Overall, 176/797 (22%) participants attended both the 6-month and the 12-month follow-ups, and 43/797 (5%) participants came back only at the 12-month follow-up (Fig. 1A). Among the 24 participants that received an HIV positive test at baseline, retention rates were 50% at 6-month follow-up, 21% at 12-month appointment and 17% came at both follow-ups (Fig. 1B).

Attending the 6-month appointment was significantly and independently associated with being followed at 12-month (adjusted OR = 4.4; 95% CI = 3.0–6.5).

The mean follow-up time for participant's 6-month and 12 -month visits was 6.6 (1.7) months and 14.8 (5.0) months, respectively. Thus, 50% of participants returned after their expected date for their 6-month follow-up and 83% returned later than expected for their 12-month follow-up.

The main sociodemographic, clinical and behavioral characteristics of participants at baseline and at 6- and 12-month follow-ups are shown in Table 1. At baseline near 60% of the participants were women and the mean age of the study population was 30 years (SD: 9). Around 70% of respondents had university studies, close to 80% reported a middle economic level, 82% were not married, 91% had a high religiosity and the great majority reported a daily/weekly access to the mobile phone (99%) and the Internet (78%). The majority of the participants perceived having a good health status (66%) and a low HIV risk (94%), most of them had been previously HIV tested (64%) and 3% got a new positive HIV diagnosis in our study. At baseline, 10% of the participants reported having had an STI diagnosis in the previous year. Regarding sexual behaviors, most participants were sexually experienced (91%), 11% reported having had sex before 15 years, around 20% had multiple sexual partners and 60, 20 and 10% reported oral, anal or paid sex, respectively. The prevalence of all these risk sexual practices was significantly lower at follow-up when paired comparisons were done at each of the follow-ups. Finally, regarding HIV-related misconceptions, at baseline 38% wrongly believed HIV is caused by witchcraft or God´s punishment. However, the percentage of participants reporting this wrong belief was significantly lower at both follow-ups.

**Table 1:** Sociodemographics, health, sexual behaviors and HIV knowledge of OKAPI participants at baseline and 6- and 12-month follow-ups and paired changes between baseline and follow-up.

	Baseline (N = 797) n (%)	6-month follow-up (N = 456) n (%)	12-month follow-up (N = 219) n (%)	0–6 month paired comparison difference (95%CI) p value* (N = 456)	0–12 month paired comparison difference (95%CI) p value* (N = 219)
**Sociodemographics**					
Sex (female)	462 (58)	268 (59)	118 (54)		
Age (mean (SD)) (years)	30 (9.4)	30 (9.3)	32 (8.9)		
15–19	40 (5)	17 (4)	1 (1)		
20–24	218 (27)	119 (26)	47 (22)		
25–59	539 (68)	320 (70)	171 (78)		
Education level					
No studies/primary studies	7 (1)	–	7 (3)		
Secondary studies	259 (32)	–	44 (19)		
University studies	531 (67)	–	168 (77)		
Perceived economic level					
Low	149 (19)	–	–	–	–
Middle	617 (77)	–	–		
High	31 (4)	–	–		
Working status					–
Unemployed/housewife	475 (60)	–	–	–	
Studying	111 (14)	–	–		
Working	211 (26)	–	–		
Civil status				–	–
Single	644 (81)	–	–		
Married	137 (17)	–	–		
Divorced	11 (1)	–	–		
Widowed	5 (1)	–	–		
Church attendance			–		–
Never/seldom/monthly	73 (9)	182 (40)			
Daily/weekly	724 (91)	274 (60)		− 0.31(− 0.4,− 0.3) < 0.001	
Media access (daily/weekly)					–
Mobile phone	789 (99)	–	–		
The Internet	626 (78)	–	184 (84)		− 0.04 (− 0.09,0.02) 0.21
**Health**					
Perceived good health status	529 (66)	353 (77)	–	0.2 (0.1,0.2) < 0.001	–
HIV risk perceived					
No/low risk	747 (94)	430 (94)	202 (92)		
Medium/high risk	50 (6)	26 (6)	17 (8)	− 0.00 (− 0.03,0.02) 0.9	− 0.00(− 0.06,0.05) 0.85
Previous HIV testing	509 (64)	–	–	–	
Attending HIV VCT^a^ with couple	125 (16)	52 (11)	10 (5)	− 0.03 (− 0.04,− 0.00) 0.02	− 0.1 (− 0.15,− 0.05) < 0.001
Intention to share an HIV + result with partner	224 (28)	–	–	–	–
Time to the closest ARV^b^ center > 60 min	147 (18)	–	–	–	–
HIV test					
Negative	747 (94)	433 (95)	209 (95)		
Positive	24 (3)	12 (3)	7 (3)	0 (− 0.00,0.00) 1.0	0 (− 0.01,0.03) 0.5
Indeterminate	26 (3)	11 (2)	3 (1)		
STI diagnosis in the last 12 months	80 (10)	–	6 (3)	–	− 0.07 (− 0.11,− 0.02) 0.002
Genital ulcer	68 (8)	–	5 (2)	–	− 0.05 (− 0.09,− 0.01) 0.01
Urinary Tract Infection	93 (12)	–	26 (12)	–	0.00 (− 0.06,0.07) 0.9
Vaginal infection	63 (8)	–	5 (2)	–	− 0.05 (− 0.09,− 0.01) 0.01
Penis infection	12 (1)	–	4 (2)	–	0 (− 0.03,0.03) 1.0
Alcohol consumption					
Never/seldom	662 (83)	372 (82)			
Weekly/daily	135 (17)	84 (18)	–	0.05 (0.01,0.08) 0.01	–
**Sexual behaviors**					
Ever sex	728 (91)	–	–	–	–
First sex before 15 years	81 (11)	–		–	–
≥ 2 sexual partners currently	134 (19)	57 (18)	13 (8)	0.03 (− 0.1,0.01) 0.12	− 0.11 (− 0.18,− 0.05) < 0.001
≥ 2 sexual partners in the previous 6 months	97 (13)	17 (5)	9 (6)	− 0.08 (− 0.1,− 0.05) < 0.001	− 0.09 (− 0.14,− 0.03) 0.003
Consistent condom use	20 (3)	16 (5)	9 (6)	0.02 (0.00,0.05) 0.03	0.02 (− 0.02,0.06) 0.48
Oral sex (ever)	430 (60)	166 (51)	30 (19)	− 0.15 (− 0.2,− 0.09) < 0.001	− 0.41 (− 0.49,− 0.33) < 0.001
Anal sex (ever)	159 (22)	47 (15)	13 (8)	− 0.11 (− 0.15,− 0.06) < 0.001	− 0.10 (− 0.17,− 0.04) 0.001
Paid sex (ever)	73 (10)	16 (5)	3 (2)	− 0.05 (− 0.07,− 0.02) < 0.001	− 0.07 (− 0.12,− 0.03) < 0.001
Forced sex (ever)					
Physical violence perpetrated by partner	218 (30)	27 (8)	10 (6)	− 0.27 (− 0.32,− 0.22) < 0.001	− 0.22 (− 0.29,− 0.15) < 0.001
Sex being afraid of partner´s reaction	114 (16)	9 (3)	–	− 0.16 (− 0.20,− 0.12) < 0.001	–
Unwanted sexual practices	94 (13)	7 (2)	–	− 0.13 (− 0.17,− 0.09) < 0.001	–
**HIV knowledge and information**					
Believe HIV is caused by witchcraft or God´s punishment	301 (38)	113 (25)	30 (14)	− 0.12 (− 0.18,− 0.07) < 0.001	− 0.19 (− 0.27,− 0.11) < 0.001
Think there is frequent HIV information in Kinshasa	148 (19)	–	–		

‘–’: variables not included in 6- or 12-month questionnaires.

^a^*VCT* Voluntary Counseling and Testing.

^b^*ARV* antiretroviral.

*McNemar test p values.

When baseline characteristics among retained and lost-to-follow-up participants were compared, at 6-month follow-up retained participants were significantly more likely to have reported being studying/working at baseline (48 vs. 30%, p < 0.001), to have a more frequent baseline use of the Internet (82 vs. 74%, p = 0.016), having a lower perception of good health (61 vs. 74%, p < 0.001), reporting genital ulcers (10 vs. 6%, p = 0.038) or urinary tract infections (14 vs. 9%, p = 0.05), having had more repeated HIV testing (68 vs. 59%, p = 0.008), having the intention to share a positive HIV test with their partner (34 vs. 20%, p < 0.001), having suffered from forced sex (37 vs. 22%, p < 0.001), living closer to ARV distribution centers (85 vs. 77%, p = 0.003), less frequently reporting common alcohol consumption (14 vs.21%, p = 0.007) and having received much information on HIV (16 vs. 23%, p = 0.0.12). At 12-month follow-up significant differences among retained and lost-to-follow-up participants were found for the frequent Internet use (88 vs. 75%, p < 0.001), repeated HIV testing (71 vs. 61%, p = 0.012), intention to share a positive result with partner (36 vs. 25%, p = 0.002) and less reporting of forced sex (11 vs. 18%, p = 0.028).

Variables significantly associated with retention in crude analyses were included in multivariate models (Table 2). Retention at 6- and 12-month follow-ups was independently associated with reporting a low economic status, being studying or working, having a daily/weekly access to the Internet, having been previously HIV tested, having the intention to share an HIV positive test with their partner and reporting forced sex. On the contrary, at 6-month follow-up, participants living far from an ARV centre, were significantly less likely to come back. Sex, age and sexual risk behaviors were not associated with retention at any follow-up.

**Table 2:** Baseline characteristics of OKAPI participants associated with retention at 6- and 12-month follow-up (logistic regression adjusted Odds Ratios).

Baseline characteristic	6 m-Retention (N = 456)	12 m-Retention (N = 219)	6- and 12 m-Retention (N = 176)	12 m-Retention considering time varying exposures
		Adjusted OR (95% CI)^a^		Adjusted OR (95% CI)^b^
**Sociodemographics**				
Sex				
Female	1.1 (0.8–1.6)1.6)	1.0 (0.7–1.5)	1.0 (0.7–1.6)	1.1 (0.8–1.4)
Male	1 (Ref.)	1 (Ref.)	1 (Ref.)	1 (Ref.)
Age (years)	1.0 (1.0–1.0)	1.0 (1.0–1.0)	1.0 (1.0–1.0)	1.0 (1.0–1.0)
Perceived economic level				
Low	2.9 (1.2–6.9)	2.9 (1.1–8.0)	2.5 (0.9–7.5)	1.6 (1.2–2.1)
High	1 (Ref.)	1 (Ref.)	1 (Ref.)	1 (Ref.)
Currently studying				
Yes	2.5 (1.5–4.2)	1.5 (0.9–2.4)	1.6 (1.0–2.7)	1.5 (1.1–1.9)
No	1 (Ref.)	1 (Ref.)	1 (Ref.)	1 (Ref.)
Currently working				
Yes	1.6 (1.0–2.3)	0.9 (0.6–1.5)	1.2 (0.7–1.9)	1.1 (0.8–1.5)
No	1 (Ref.)	1 (Ref.)	1 (Ref.)	1 (Ref.)
Frequency of access to the Internet				
Weekly/daily	1.8 (1.2–2.6)	2.7 (1.6–4.5)	2.9 (1.7–5.0)	1.9 (1.4–2.5)
No	1 (Ref.)	1 (Ref.)	1 (Ref.)	1 (Ref.)
**Health**				
Perceived health status				
Good	0.6 (0.4–0.9)	1.0 (0.7–1.5)	0.9 (0.6–1.3)	1.3 (1.0–1.6)
No	1 (Ref.)	1 (Ref.)	1 (Ref.)	1 (Ref.)
Previous HIV tests				
Yes	1.6 (1.1–2.2)	1.5 (1.1–2.1)	1.5 (1.0–2.2)	1.3 (1.1–1.7)
No	1 (Ref.)	1 (Ref.)	1 (Ref.)	1 (Ref.)
Intention to share HIV test result with partner				
Yes	1.8 (1.2–2.7)	1.7 (1.2–2.6)	1.8 (1.2–2.8)	1.6 (1.2–2.1)
No	1 (Ref.)	1 (Ref.)	1 (Ref.)	1 (Ref.)
Time to the closest ARV center				
> 60mins	0.5 (0.4–0.8)	0.7 (0.5–1.1)	0.9 (0.5–1.4)	0.7 (0.5–0.9)
< 15mins	1 (Ref.)	1 (Ref.)	1 (Ref.)	1 (Ref.)
Alcohol consumption				
Daily/weekly	0.6 (0.4–0.9)	0.8 (0.5–1.3)	0.8 (0.5–1.3)	0.8 (0.6–1.1)
No	1 (Ref.)	1 (Ref.)	1 (Ref.)	1 (Ref.)
Victim of unwanted sexual practices				
Ever	1.7 (1.0–2.7)	0.6 (0.3–0.9)	0.8 (0.5–1.4)	3.4 (2.2–5.2)
Never	1 (Ref.)	1 (Ref.)	1 (Ref.)	1 (Ref.)
**HIV information**				
Thinks there is frequent HIV information in Kinshasa				
Yes	0.6 (0.4–0.9)	1.3 (0.8–1.9)1.3 (0.8–1.9)	1.2 (0.8–1.9)	0.8 (0.6–1.1)
No	1 (Ref.)	1 (Ref.)	1 (Ref.)	1 (Ref.)

^a^Multivariate logistic regression including all variables in the table and the corresponding Odds Ratios and their 95% confidence intervals.

^b^Multivariate logistic regression including all variables in the table and the corresponding Odds Ratios and their 95% confidence intervals (Generalized Estimated Equation).

## Discussion

Research on retention in HIV studies in SSA has been focused on people living with HIV within the HIV continuum of care. Fewer studies have analyzed the retention rates and associated factors in observational cohorts. In this prospective HIV cohort including people attending HIV Voluntary Counseling and Testing in Kinshasa, and having both positive and negative tests, low retention rates were found: 57% at the 6-month follow-up appointment and 27% at the 12-month follow-up, with only 22% of the participants completing the two scheduled visits. Among participants who got an HIV positive test result, retention rates were 50% and 21% at 6- and 12-month visits, respectively.

Data on retention rates in the DRC come from retrospective cohorts including patients living with HIV within HIV care and ARV programs^29,32,35–37^. They all show higher retention rates than ours, varying from 67 to 96%. Recent studies from different countries in SSA show retention rates between 17 and 83% after 12 months^10,15,23,31,38–40^. Most of these studies were conducted among people HIV positive involved in HIV care programs, which makes them more motivated to continue in the study. On the contrary, most of our participants were HIV negative, with no need of coming back to healthcare. In an Ugandan cohort that evaluated factors associated with dropout among high-risk participants invited for HIV testing and having a negative result, researchers found that 92% were followed up after 6 months, 85% at 12 months and 76% at 18 months^19^, thus, retention rates much higher than those observed in our study in Kinshasa were found. This was probably because they were invited for HIV screening (compared to the client-initiated VCT in our cohort), they were high-risk individuals which may have moved them to attend follow-up visits, they included more home visits and also because in the study condoms and medical care including STIs diagnosis and treatment were offered to them. In another study published by the same research group they analyzed retention among high-risk women with an HIV negative test and they found that over the 18-month follow-up period, 93% came back at least once^41^. The higher retention observed may have been influenced by the fact that previous to the screening participants attended community meetings in which information about the study was provided. Also, participants compliant with study follow-ups were trained as leaders that reminded other participants to attend their visits and reported about participants who had migrated. Finally, the same research team published another study that similarly evaluated retention among high-risk HIV negative people in the same Ugandan fishing communities. It was an HIV vaccine preparedness study in which door-to-door HIV counseling and testing was offered. Over the 24-month follow-up period, retention was 70%^42^. Another HIV vaccine trial preparedness cohort in South Africa found a 85% retention among participants who were HIV negative. Factors that probably contributed were the group workshops on HIV and vaccines hold along the study period and the HIV, syphilis and pregnancy tests performed at each 3-month follow-up visits^43^. Mutagoma et al. found a 92% retention rate after 12-month follow-up among participants in the Rwandan AIDS indicator and HIV incidence longitudinal household survey. The authors discussed factors that helped to have good retention rates such as the small size of Rwanda which facilitates transportation and the knowledge about people relocations and migrations among community members and the support of the national media^23^. Conley and cols. evaluated retention in a prospective cohort study among HIV-1 serodiscordant heterosexual couples and found a retention around 80%. All couples received at least one home visit, support groups were available, trimethoprim-sulfamethoxazole prophylaxis was supplied for partners HIV-infected, free drinks were offered during clinic visits, and transportation fees to and from the clinic were covered^44^.

Considering the retention observed in other African cohorts and that rates of follow-up around 80% have been recommended in cohort studies^45^, more work is needed to monitor and improve retention in Kinshasa, working on different factors that assure better follow-ups in cohort studies or clinical trials.

In our study significant associations were not found for participants´ age and sex, although most studies show that retention rates increase with age and among females^12,19,46–49^. In our cohort, education level was neither a predictive factor of better retention. Most of the evidence coming from SSA studies show that having no education or lower levels of education is associated with higher attrition rates^5,12,15,23,40,47,50^, with the exception of one study in which patients with secondary or higher education were more frequently LTFU^32^. In our cohort being a student (89% of them university students) was associated with higher retention at 6-month follow-up, probably due to being more responsible or sensitive with research. Also, participants working at baseline were more likely to be retained at 6-month follow-up compared to unemployed participants, although no significant associations were found for 12-month follow-up and for those attending both follow-up appointments. Other studies have found that working is associated with higher attrition rates, probably as a result of inflexible work schedules^19,28,50,51^. However, some authors have found opposite results, with lack of formal employment being a barrier to retention^33,52^. Within HIV care, higher lost-to-care rates have been found among patients with a paid profession maybe as a result of being more able to transfer to a private clinic^5^.

Regarding economic level, literature shows that reporting a lower socioeconomic status, family financial constraints or perceiving social class as poor are associated with being LTFU^15,28,30,52,53^. In our study participants with a low economic level were significantly more likely to return at 6- and 12-month follow-ups. This may be due to the fact that all participants had a monetary incentive for transportation at follow-up, which probably motivated them to come back to the appointments. The same effect of economic incentives for transportation has been shown by other authors^54,55^. In fact, a key factor affecting retention rates are transportation costs or lack of transportation^15,24,28,30,33,46,50,52,56^. Teague and colleagues carried out a systematic review in 2018 including 143 longitudinal cohort studies to assess the effectiveness of different retention strategies and found that `barrier-reduction´ strategies were associated with higher retention^27^. Related to transportation barriers, reporting a longer distance to the closest center for ARV distribution was inversely associated with retention at 6-month follow-up, as previously reported by other authors^4,15,25,30,52,57^. As recommended by other researchers^27,46^, home visits were offered to improve retention in the cohort. Although some LTFU participants were retained, home visits were not an easy nor efficient strategy in our study.

With respect to the perceived health status, as shown by other authors^25^, participants perceiving they had a good health were less likely to attend the 6-month follow-up visit (significance was lost for the 12-month follow-up). It makes sense that people with good health do not perceive the need to be followed up.

Participants previously HIV tested were significantly more likely to attend follow-ups. As shown by other authors^23,58^, the wish to know one´s serostatus encourages people to participate in studies and to continuously want to be tested. However, other authors have shown that perceiving that an HIV negative test will not change, may be a barrier for repeating HIV testing^59^, which could explain why many of the participants in our cohort, who were mostly HIV negative, did not return for the follow-up visit. In this sense, some authors suggest HIV pre-exposure prophylaxis (PrEP) availability and provision could be a potential incentive to improve retention among people attending HIV testing^60,61^. In the DRC the knowledge and access to the PrEP are still low today^61,62^, adherence to antiretrovirals is not high^63^ and ARV resistance is present^64^. Therefore, it does not seem to be an option today in the country although it might be an option to be considered in the future for high risk population attending HIV testing.

Regarding disclosure of HIV status, higher retention rates were found among participants that had the intention to share a positive HIV test result with their partner; this was consistent with other studies^33,46,50,52^. The intention of non-disclosure is more frequent in patients perceiving or fearing stigma, which may cause participants to miss their appointments^12,30,46,52,65^. In fact, among women, fear of repercussions from their partners has been associated with attrition^15,65^.

No significant associations were found between sexual risk behaviors and retention. Some studies have shown that having multiple sexual partners or using condoms were associated with participant dropouts^12,19^, however, there is few evidence on this.

Retention at 6-month follow-up was lower for those participants thinking there was frequent HIV information in Kinshasa. They probably did not find it necessary to contact the healthcare center again, once they were HIV tested and counseled. Other studies have shown an association between poor knowledge on STI or HIV and being LTFU^12,52^, which can be a result of not perceiving the infections negative consequences.

Despite the repeated reminder phone calls in our study a low retention was found. People in Kinshasa, as well as in other SSA, frequently lose or change their phone numbers^28,50^. Likewise, Teague and colleagues showed that retention was significantly higher in studies not including phone call reminders compared to those with phone call reminders. In our study, reporting a weekly/daily access to the Internet at baseline was significantly associated with a better retention at 6-month, 12-month as well as 6- and 12-month follow-ups. Considering that the access to the Internet is increasing in Kinshasa (as observed among our participants most of whom reported a frequent access), other innovative approaches could be a good option for maintaining regular contacts and participants´ motivation and improve retention, as proposed by different authors^11,28,66,67^. There is few evidence on new strategies based on modern technology that need to be evaluated in future studies to improve participants´ retention.

Based on the factors that have been found to be associated with better retention in our cohort, and taking into account Teague and cols´ findings for their four proposed groups of retention strategies (*barrier-reduction*; *community-building*; *follow-up strategies (eg. incentives)/reminders*; and *tracing)*, it can be highlighted that a better design of home visits and of alternative methods of data collection for barrier-reduction is needed. According to Teague and cols, *barrier-reduction* strategies, such as home visits, and *community-building* strategies (i.e. emphasizing the benefits of the study, giving feedback and sending thank you and birthday messages to the participants) were the most efficient in lowering attrition^27^. This could similarly help improve retention in cohorts in the DRC. Regarding *follow-up/reminders* strategies, the different options they analyzed did not show to improve retention, neither did *tracing* strategies, such as the tracing via alternative contacts that was considered in our cohort. They did not improve retention. Finally, the different Internet options within all different retention approaches need to be evaluated.

Our study has some limitations that need to be considered. First, the reasons for being LTFU were not well documented and information regarding death, migration or other physical or psychological diseases, described in other SSA studies^3,29^ was lacking. Considering that the vast majority of our participants tested HIV negative, a high mortality rate is quite unlikely in our cohort. Other reasons such as going to other health facilities for new HIV/STI testing, the distance to our hospital or the presence of other comorbidities do need to be taken into account in future studies. Secondly, considering the 100% response rate observed in our project, there seems to be a possibility that agreeing to participate was a polite and socially desirable answer, possibly explaining the low retention rates in our cohort.

Despite these limitations, our study has some strengths that need to be highlighted. This is the first study in the DRC evaluating the retention rates and associated factors in an HIV research cohort including participants attending HIV Voluntary Counseling and Testing and not in the continuum of HIV care and treatment. Secondly, 797 participants were included in the cohort, a sample size that allowed evaluating multiple factors using multivariate analyses and that can help the local policymakers to improve retention in different HIV research studies in the country.

In conclusion, our results show a high attrition among people attending HIV VCT and participating in a prospective cohort in Kinshasa. Considering the factors associated with a better retention, such as the economic incentive at follow-up, the repeated HIV test or the use of the Internet, this study underscores the need of designing and implementing new strategies to improve retention in HIV research studies in Kinshasa.
